# CANT1 serves as a potential prognostic factor for lung adenocarcinoma and promotes cell proliferation and invasion in vitro

**DOI:** 10.1186/s12885-022-09175-2

**Published:** 2022-01-28

**Authors:** Qiwei Yao, Yilin Yu, Zhiping Wang, Mengyan Zhang, Jiayu Ma, Yahua Wu, Qunhao Zheng, Jiancheng Li

**Affiliations:** Fujian Medical University Cancer Hospital, Fujian Cancer Hospital, Fuzhou, 350014 China

**Keywords:** CANT1, Lung adenocarcinoma, TCGA, Prognosis, Methylation

## Abstract

**Background:**

Calcium-activated nucleotidase 1 (CANT1), functions as a calcium-dependent nucleotidase with a preference for UDP. However, the potential clinical value of CANT1 in lung adenocarcinoma (LA) has not been fully clarified. Thus, we sought to identify its potential biological function and mechanism through bioinformatics analysis and in vitro experiments in LA.

**Methods:**

In the present study, we comprehensively investigated the prognostic role of CANT1 in LA patients through bioinformatics analysis and in vitro experiments. The Cancer Genome Atlas (TCGA) and Gene Expression Omnibus (GEO) were utilized to analyze the expression of CANT1 in LA patients and their clinical-prognostic value. The immunohistochemistry staining was obtained from the Human Protein Atlas (HPA). A Cox regression model was used to evaluate prognostic factors. Gene ontology (GO) and Gene set enrichment analysis (GSEA) was performed to explore the potential regulatory mechanism of CANT1 in the development of LA. Moreover, we also examined the relationship between CANT1 expression and DNA methylation. Finally, we did in vitro experiments to evaluate the biological behavior and role of CANT1 in LA cells (LACs).

**Results:**

Our study showed that the CANT1 expression was significantly elevated in the LA tissues compared with the normal lung tissues. Increased CANT1 expression was significantly associated with the TN stage. A univariate Cox analysis indicated that high CANT1 expression levels were correlated with poor overall survival (OS) in LA. Besides, CANT1 expression was independently associated with OS in multivariate analysis. GO and GSEA analysis showed the enrichment of mitotic nuclear division, DNA methylation, and DNA damage. Then we found that the high expression of CANT1 is positively correlated with hypomethylation. The methylation level was associated with prognosis in LA patients. Finally, in vitro experiments indicated that knockdown of CANT1 resulted in decreased cell proliferation, invasion, and G1 phase cell-cycle arrest in LACs.

**Conclusion:**

The present study suggested that CANT1 may serve as a potential prognosis biomarker in patients with LA. High CANT1 expression and promoter demethylation was associated with worse outcome. Finally, in vitro experiments verified the biological functions and behaviors of CANT1 in LA.

## Background

Lung cancer is the leading cause of cancer death and the second most common cancer in both men and women [1]. Among them, the incidence of lung adenocarcinoma (LA), the most common histological type of lung cancer, is increasing year by year [2–4]. Despite immense progress in therapeutic strategies, including surgery, molecular targeted agents, and immune checkpoint inhibitors, the 5-year survival rate among patients with non-small cell lung cancer (NSCLC) is less than 20% [5–7]. The high mortality for NSCLC patients may be driven by two factors: one is that about 66% of NSCLC patients are diagnosed at a relatively advanced stage. The other one is that even in the early-stage portion, the mortality rate is still very high because of the high recurrence rate after surgery [8, 9]. Therefore, there is an urgent need to identify and develop novel molecular markers to improve the prognosis of LA.

Calcium-activated nucleotidase 1 (CANT1), also known as DBQD, SCAN-1, SCAN1, SHAPY, functions as a calcium-dependent nucleotidase with a preference for uridine diphosphate (UDP). The protein encoded by CANT1 belongs to the apyrase family. CANT1 is generally expressed in human tissues [10]. Alternative splicing transcript variants have been noted for this gene. The alternative splicing transcript variants often appear in cancer and have been regarded as an important sign of tumor progression and cancer treatment [11]. Cancer cells often show abnormal alternative splicing profiles to produce isoforms, stimulating cell proliferation and migration or increasing apoptosis resistance [12]. CANT1 is a new oncogenic mRNA in some cancer types, like human clear cell renal cell carcinoma (ccRCC) and prostate cancer. These studies showed that CANT1 was associated with cancer cell proliferation, migration, and invasion. Besides, the high expression of CANT1 was positively correlated with TNM stage and histological grade in ccRCC [13, 14]. In addition, some studies have indicated that mutations in CANT1 are associated with abnormal growth and development [15, 16]. Previous research has shown that factors related to normal growth often play a momentous role in tumor growth [17]. These results suggest the potential role of CANT1 as a prognostic biomarker in cancer. Although CANT1 plays a crucial role in some types of tumors, it is unclear whether it is a prognostic factor of LA. Besides, there is little literature about the relationship between CANT1 and LA reported so far. It is the rare study to demonstrate the functional mechanism of CANT1 in LA.

CANT1 is a phosphatase whose preferred substrate is UDP and followed by GDP, UTP, GTP, ADP, and ATP [10]. Gerhardt et al. indicated a G1 phase cell-cycle arrest on CANT1 knockdown in LNCaP and PC-3 cells [13]. Another result showed that knockdown of CANT1 induced an S-phase cell-cycle arrest, resulting in a decrease in the number of cells entering the G2/M-phase [14]. It also demonstrated that CANT1 knockdown could promote apoptosis. Several genes involved in the apoptotic pathways were altered after CANT1 was knocked out in 769-P cells. Such as the pro-apoptotic factors of BAD and cleaved caspase-3 were upregulated, while the anti-apoptotic factor of BCL2 was downregulated. Serine, glycine, threonine metabolism, purine metabolism, and glycosphingolipid biosynthesis in the genes’ pathway are significantly altered [14]. Besides, CANT1 might contribute to tumorigenesis via activating both MAPK and NF-kB signaling pathways [18]. However, little literature has explored the association between CANT1 and LA. Therefore, our study aimed to elucidate the expression of CANT1 and its potential value of therapy and prognosis in LA tissues.

In the present study, we comprehensively investigated the prognostic role of CANT1 in LA patients through bioinformatics analysis and in vitro experiments. This research aimed to identify CANT1 gene with potential prognostic relevance in patients with LA. Up to now, there is no evidence that CANT1 relates to the occurrence and development of LA. Therefore, our study aimed to elucidate the expression of CANT1 and its potential value of therapy and prognosis in LA tissues. Besides, there is little literature about the relationship between CANT1 and LA reported so far. It is the rare study to demonstrate the functional mechanism of CANT1 in LA. In the study, we explored the effect of CANT1 expression and methylation on prognosis to determine whether CANT1 can be a potential prognostic biomarker for patients with LA. Finally, we also verified the biological function and behavior of CANT1 in LACs through in vitro experiments. This further strengthens our understanding of the biological mechanism of CANT1.

## Methods

### Clinical information from TCGA and GEO databases

The lung adenocarcinoma tissues and adjacent tissues included in our study were from the TCGA database, where a total of 502 LA patients from 1991 to 2013. Gene expression data and the corresponding clinical information of the TCGA-LA were obtained from the TCGA (https://portal.gdc.cancer.gov/). The GEO databases of GPL570 and GPL6884 platforms were performed for the validation (www.ncbi.nlm.nih.gov/geo). Expression profiles (HTSeq-Counts) were compared between high and low CANT1 expression groups to identify differentially expressed genes (DEGs) using the DESeq2 R package. The data used in this study were in accordance with the guidelines provided by TCGA and GEO. The ethics approval and informed consent were not required.

### Over-expression of CANT1 in LA patients

We explored the expression differences of CANT1 in LA patients between tumor tissue and normal tissue. Besides, the immunohistochemistry staining of CANT1 expression was obtained by the Human Protein Atlas (HPA) (https://www.proteinatlas.org) [19, 20]. On the HPA website, antibodies HPA019627, HPA022818, and HPA019639 were used for immunohistochemistry staining of CANT1.

### Role of CANT1 in LA patient’s survival

A total of 502 LA patients were included in our study. The survival curve was performed by survival R package and survminer R package. Furthermore, we validated the prognostic values of the risk model in two independent datasets from GSE31219 (*n* = 83) and GSE41271 (*n* = 181).

### Construction and evaluation of the nomogram model

A nomogram was built based on the multivariate analysis’s result to predict the survival probability for 1-year, 3-years, and 5-years of LA patients. The rms R package was performed to produce a nomogram. The concordance index (C-index) and calibration curve were performed by the Hmisc R package. In this study, C-index was carried out to determine the nomogram’s discrimination with 1000 bootstrap replicated.

### Functional enrichment analysis

GEPIA is a database based on the UCSC Xena project [21]. In the study, GEPIA 2.0 (http://gepia2.cancer-pku.cn/) was performed to find the gene with the most similar expression of CANT1 in LA. Finally, 50 most correlated co-expression genes were founded. Then the functional analysis of CANT1 and its co-expression genes in TCGA-LA were predicted by Metascape (http://metascape.org) [22].

### Gene set enrichment analysis

Gene set enrichment analysis (GSEA) is a calculation method that determines whether a set of prior defined genes show statistically significant and consistent differences between two biological states [23, 24]. In this study, GSEA was performed using the Molecular Signatures Database (MSigDB) Collection (c2.all.v7.0.entrez.gmt) of clusterProfiler R package to illuminate the statistically significant pathway difference between high and low CANT1 expression groups of LA. The expression level of CANT1 was used as a phenotype label. The pathway terms with adjusted *P*-value < 0.05 and false discovery rate (FDR) *q*-value < 0.25 were considered significantly enriched.

### Correlation between CANT1 expression and DNA methylation

The previous GSEA analysis suggested that CANT1 was related to DNA methylation. Therefore, we then analyzed the relationship between the expression of CANT1 and DNA methylation. Spearman correlation coefficient was performed to reveal the correlation between the expression of CANT1 and methylation level in TCGA-LA. The gene methylation data of the TCGA-LA project was obtained from the UCSC Xena browser (version:07-20-2019, https://xenabrowser.net/datapages/). The relationship between the methylation level and overall survival (OS) of CANT1 was retrieved from MethSurv (https://biit.cs.ut.ee/methsurv/). It is a web tool to provide univariable and multivariable survival analysis based on DNA methylation biomarkers by using TCGA data [25].

### Cell lines and cell culture

Human lung adenocarcinoma cell lines A549 and H1299 were obtained from the Genechem company (Shanghai, China). A549 cells were cultured in Ham’s F-12 K medium (PYG0036, Boster, USA) supplemented with 10% fetal bovine serum (FBS, P30-3302, PAN biotech, Germany) and 1% penicillin/streptomycin (100x, Gibco, USA), H1299 Cells were cultured in RPMI-1640 (PYG0006, Boster, USA) supplemented with 10% FBS and 1% penicillin/streptomycin. All cell lines were maintained in a humidified incubator with 5% CO2 at 37 °C. The medium was changed every 3 days, and a subcultivation ratio of 1:3 was performed.

### Small interfering RNAs transfection

Small interfering RNAs (siRNAs) were used to selectively knockdown the expression of CANT1 by the Lipofectamine 3000 transfection reagent (L3000-015, Invitrogen, USA), according to the manufacturer’s instructions. The sequence of three groups of siRNAs targeting CANT1 (siRNA-987, siRNA-1173, and siRNA-273) and two negative control groups (si-NC and NC) were obtained from the zolgene (Fuzhou, China) shown in Table 1. For transfection, A549 and H1299 cells in the logarithmic growth phase were inoculated into a six-well plate. Knockdown efficiency was verified on the RNA level using quantitative real-time polymerase chain reaction (qRT-PCR).

**Table 1:** The sequence of three groups of siRNAs targeting CANT1 (siRNA-987, siRNA-1173, and siRNA-273) and negative control group (si-NC)

siRNA Name	Sequence (5’to 3′)	Length	GC%
CANT1(human)siRNA-987	GGGUGAAGGUGGUGGGCUATT	21	57.1
CANT1(human)siRNA-987	UAGCCCACCACCUUCACCCTT	21	57.1
CANT1(human)siRNA-1173	AGAAGGACGACGAGCGCAATT	21	52.4
CANT1(human)siRNA-1173	UUGCGCUCGUCGUCCUUCUTT	21	52.4
CANT1(human)siRNA-273	CGGAAUGGAAUGAGUCUAUTT	21	38.1
CANT1(human)siRNA-273	AUAGACUCAUUCCAUUCCGTT	21	38.1
Negative control	UUCUCCGAACGUGUCACGUTT	21	47.6
Negative control	ACGUGACACGUUCGGAGAATT	21	47.6

*siRNAs* Small interfering RNAs, *NC* Negative control, *CANT1* Calcium-activated nucleotidase 1

### Quantitative real-time polymerase chain reaction

Total RNA was isolated using the TRIzol™ Reagent (15,596,026, Invitrogen, USA). Then the GoScript™ Reverse Transcription System (A5001, Promega, USA) was employed to reversely transcribe RNA into cDNA. qRT-PCR was conducted using UltraSYBR Mixture (CW0957, Cwbio, China) to measure the expression of CANT1. β-actin was used as the internal reference. The calculation of inhibitory efficacy of the CANT1 mRNA was calculated using the 2^-ΔΔCt^ method. The sequences of primers used in qRT-PCR were shown in Table 2.

**Table 2:** The sequences of primers used in qRT-PCR

Primer	Sequence (5’to 3′)	Length
β-actin(Homo)	TGACGTGGACATCCGCAAAG	20
β-actin(Homo)	CTGGAAGGTGGACAGCGAGG	20
CANT1-F(Homo)	GCTTCTCGTCCTTCAAGTTCATC	23
CANT1-R(Homo)	ACGCTTCCGATCTTGGTCTC	20

*qRT-PCR* Quantitative real-time polymerase chain reaction, *CANT1* Calcium-activated nucleotidase 1

### Cell counting kit-8 (CCK-8) assay

After transfection, we used the CCK-8 reagent (40203ES60, Yeasen, Shanghai, China) to measure cell viability. The si-CANT1 group, si-NC group, and NC group of A549 and H1299 cells were subcultured in a 96-well plate and then incubated for 0, 24, 48, and 72 h. Before detection, 10 μl (10%) CCK8 reagent was added daily to each hole in the 96-well plates and incubated at 37 °C for 1 h. Then, a microplate reader (Nano-100, Allsheng, Hangzhou, China) was used to measure the absorbance at 450 nm.

### Cell invasion assay

The invasive ability of the A549 and H1299 cells was assessed with Transwell chambers and Matrigel. The Transwell chambers were coated with 50 μl diluted Matrigel (1:8) at 37 °C for 4 h. Subsequently, 1 × 10^5^/mL cells resuspended in serum-free medium were added into the Transwell chamber, which was placed in cell culture medium supplemented with 10% FBS and was cultured for 48 h. Then the residual cells in the upper chamber were wiped with cotton swabs. Cells migrated through the membrane were fixed with 4% paraformaldehyde (P1110, Solarbio, Beijing, China), washed with PBS, mixed with 0.4% crystal violet solution (C0121, Beyotime, Shanghai, China), and counting in 3 randomly picked view under a microscope at 100× magnification (MF53, Mshot, Guangzhou, China).

### Flow cytometry

For cell cycle detection, 1 × 10^6^/ml cells were counted and washed with phosphate buffer saline (PBS, ABA212278, Hyclone, USA), followed by the addition of 70% cold ethanol at 4 °C overnight. After washing with cold PBS, the fixed-cell pellets were collected by centrifugation and resuspended in ribonuclease (RNase) A/Propidium Iodide (PI) staining buffer. The cell-cycle distribution was analyzed by flow cytometer (FACSVerse, BD, USA).

### Statistical analysis

All statistical analysis was performed using SPSS (version 26.0) and R (version 4.0.2). Wilcoxon rank-sum test and Wilcoxon signed-rank test were used to compare the expression of CANT1 between LA and normal groups. X-tile software (version 3.6.1, https://medicine.yale.edu/lab/rimm/research/software/) was performed to seek the optimal cutoff value of CANT1 expression. The relationship between the TN stage and the expression of CANT1 was analyzed with Wilcoxon single-rank test. Clinical parameters (age, gender, number pack years smoked, epithelial growth factor receptor (EGFR) mutation status, kirsten rat sarcoma viral oncogene (KRAS) mutation status, anaplastic lymphoma kinase (ALK) mutation status, T stage, N stage, M stage, pathological stage, and gene expression) associated with OS in LA patients were evaluated with Cox analyses. In univariate analysis, all factors with *P* < 0.20 were involved in multivariate analysis to identify independent prognostic factors. Owing to missing data exceeding 20%, the ALK mutation status and M stage were not included in multivariate analysis. Comparisons between two groups were analyzed by t-test. All experiments were performed with at least three independent replications. All tests were two-sided, and a *P*-value < 0.05 was considered statistically significant.

## Results

### Clinical information from TCGA database

The clinical data including 502 patients were collected from TCGA (Table 3).

**Table 3:** TCGA lung adenocarcinoma patient characteristics

Clinical characteristics	Total (502)	Percentage (%)
Gender		
Male	232	46.2%
Female	270	53.8%
Age		
< =70 years old	330	65.7%
> 70 years old	162	32.3%
Missing	10	2.0%
Number pack years smoked		
< =40	362	72.1%
> 40	140	27.9%
EGFR mutation status		
Yes	81	16.1%
No	191	38.0%
Missing	230	45.9%
KRAS mutation status		
Yes	63	12.5%
No	245	48.8%
Missing	194	38.7%
ALK mutation status		
Yes	35	7.0%
No	207	41.2%
Missing	260	51.8%
T stage		
T1	168	33.5%
T2	268	53.4%
T3	45	9.0%
T4	18	3.6%
Missing	3	0.6%
N stage		
N0	326	64.9%
N1	94	18.7%
N2	69	13.7%
N3	2	0.4%
Missing	11	2.2%
M stage		
M0	333	66.3%
M1	24	4.8%
Missing	145	28.9%
TNM stage		
Stage I	270	53.8%
Stage II	119	23.7%
Stage III	80	15.9%
Stage IV	25	5.0%
Missing	8	1.6%
CANT1 expression		
Low	210	41.8%
High	292	58.2%
Vital status		
Dead	182	36.3%
Alive	320	63.7%

*EGFR* Epithelial growth factor receptor, *KRAS* Kirsten rat sarcoma viral oncogene, *ALK* Anaplastic lymphoma kinase, *T* Tumor, *N* Regional lymph node, *M* Metastasis, *CANT1* Calcium-activated nucleotidase 1

### Over-expression of CANT1 in LA patients

**Fig. 1:**
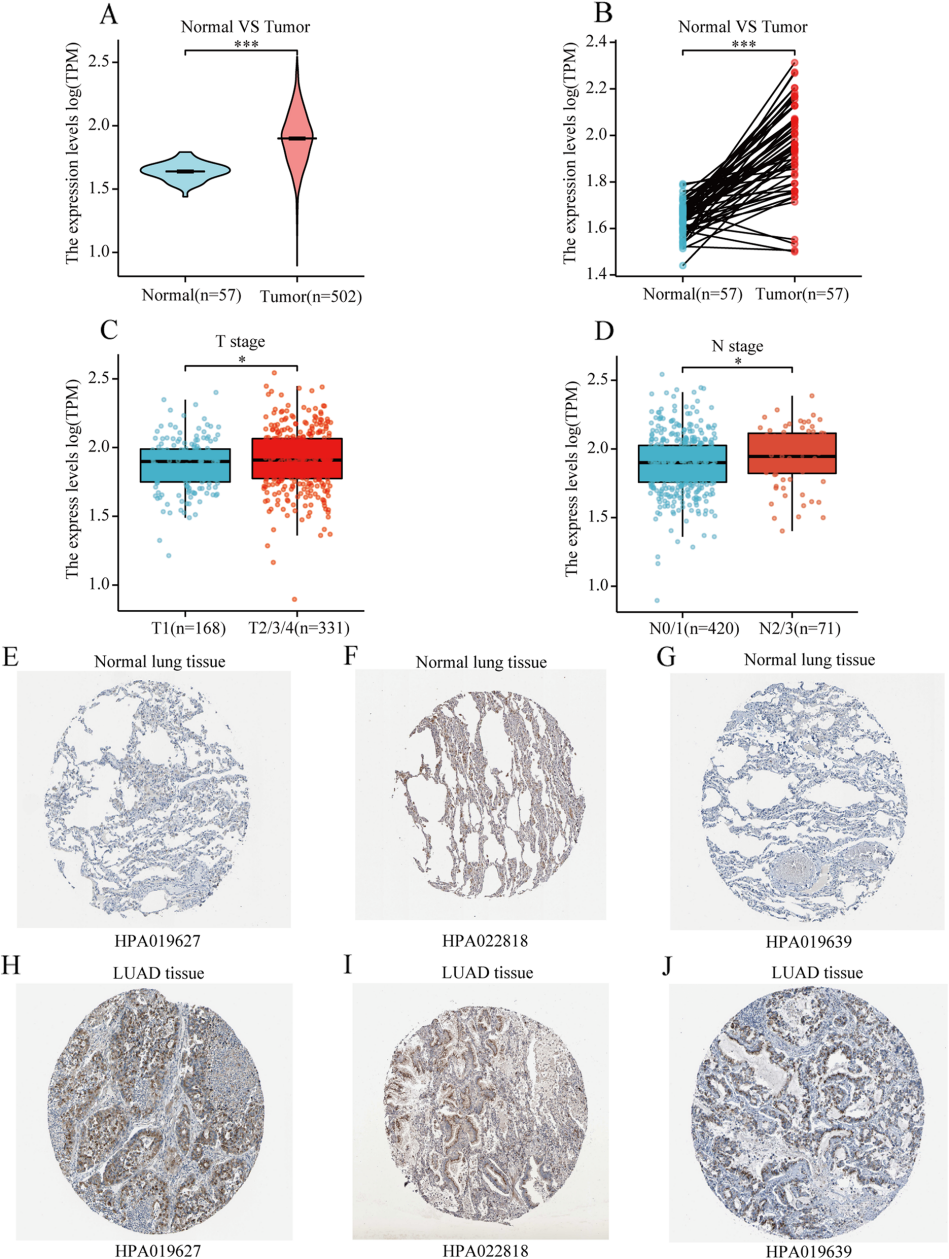
The expression levels of CANT1 in LA from TCGA data. **A** Expression levels of CANT1 in non-paired tumor and normal samples; **B** Expression levels of CANT1 in paired tumor and normal samples; **C**, **D** Association between T stage, N stage, and the expression of CANT1; **E**-**J** The expression levels of CANT1 in LA were obtained from the HPA. (*P < 0.05, **P < 0.01, ***P < 0.001). CANT1, calcium-activated nucleotidase 1; LA, lung adenocarcinoma; TCGA, The Cancer Genome Atlas; T, tumor; N, regional lymph node; HPA, Human Protein Atlas

We used the Wilcoxon rank-sum test to investigate the expression of CANT1 in 502 LA tissues and 57 normal tissues. The expression of CANT1 was significantly higher in LA groups than in normal groups (*P* < 0.001) (Fig. 1A). In addition, we further used Wilcoxon singed-rank test to examine the expression of CANT1 in 57 pairs of LA tissues. The result showed CANT1 was significantly overexpressed in LA (*P* < 0.001) (Fig. 1B). As shown in Fig. 1C-D, increased CANT1 expression in LA was significantly associated with the TN stage (T1 vs. T2/T3/T4, *P* = 0.042; NO/ N1 vs. N2/N3, *P* = 0.041). We also carried out an analysis of the association between CANT1 expression and M (metastasis) status. Due to the result are negative and meaningless, we did not add this result to the Figure. These results demonstrated that the CANT1 expression level could affect LA patients’ prognosis with different TN stage. After exploring the CANT1 expression patterns of CANT1 in LA, we tried to examine the protein expression patterns of CANT1 in LA by the HPA. As shown in Fig. 1E-J, low protein expressions of CANT1 were observed in normal lung tissues, while high protein expressions were detected in LA tissues. The staining intensity was scored as negative, weak and negative in HPA019627, HPA022818, and HPA019639 in normal lung tissues. While the staining intensity was scored as Strong in HPA019627, HPA022818, and HPA019639 in LA tissues. The quantity was scored as none, < 25%, and none in HPA019627, HPA022818, and HPA019639 in normal lung tissues. While the quantity was scored as > 75, > 75, and 25%-75% in HPA019627, HPA022818, and HPA019639 in LA tissues.

### Role of CANT1 in LA patient’s survival

**Fig. 2:**
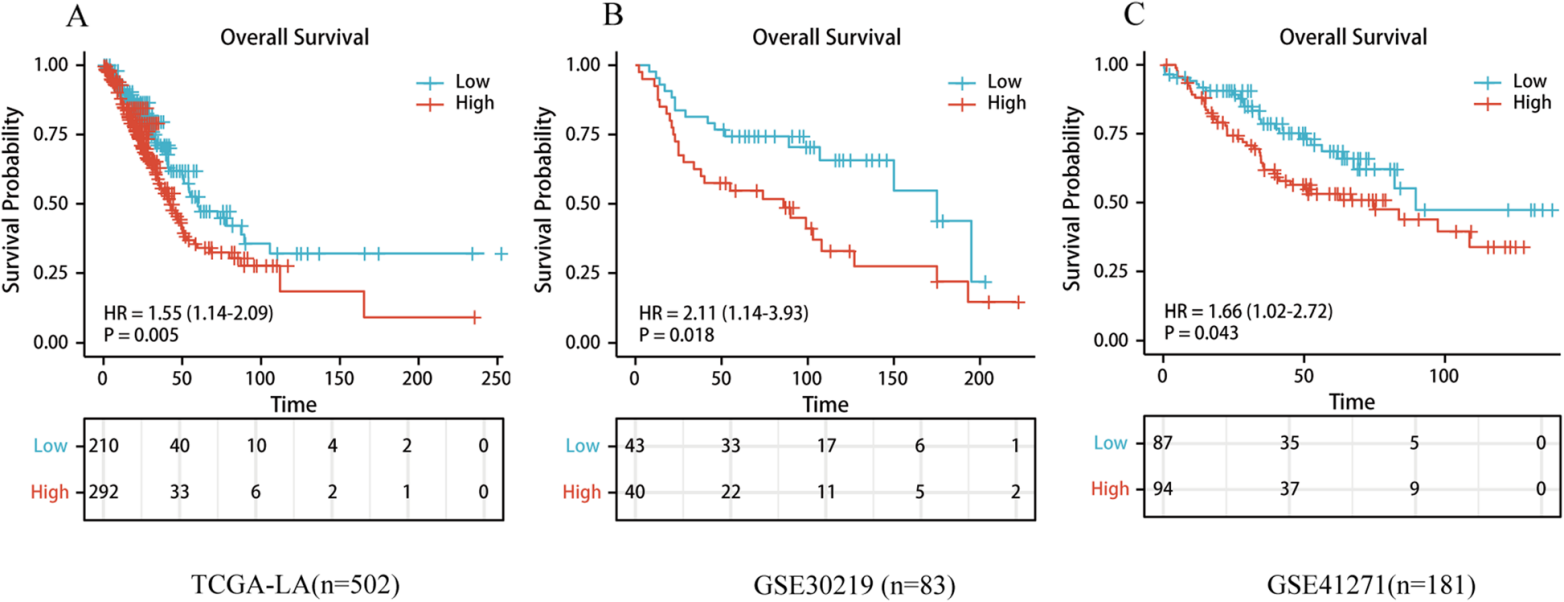
The prognostic value of CANT1 expression in LA. **A** Survival curves of OS from TCGA data (*n* = 502); **B**, **C** Survival curves of OS from GSE30219 and GSE41271 data (*n* = 83 and 181, respectively). CANT1, calcium-activated nucleotidase 1; LA, lung adenocarcinoma; OS, overall survival; TCGA, The Cancer Genome Atlas

Overall survival (OS) was significantly reduced in patients with high CANT1 expression than those with low CANT1 expression (*P* = 0.005) (Fig. 2A). To validate the association between the expression of CANT1 and OS, we also performed the GSE30219 and GSE41271 datasets from the GEO. The outcome indicated that OS was significantly reduced in patients with high CANT1 expression than those with low CANT1 expression (*P* = 0.018, *P* = 0.043, respectively) based on GSE30219 and GSE41271 database (Fig. 2B-C). We further used the univariate Cox regression model to identify prognostic factors in LA (Table 4). The result showed that high CANT1 expression levels were correlated with worse OS (*P* = 0.005, hazard ratio [HR] = 1.547 (95% confidence interval [CI] [1.143-2.094])). In addition, age > 70 years old (*P* = 0.013, HR = 1.464(95% CI [1.083-1.979]), higher TNM stage (T: *P* = 0.004, HR = 1.666 (95%CI [1.182-2.347]); N: *P* < 0.001, HR = 2.265 (95% CI [1.584-3.239]); M:*P* = 0.006, HR = 2.143 (95% CI [1.251-3.672])), and higher pathological stage (*P* < 0.001, HR = 2.609 (95%CI [1.911-3.561])) were also associated with poor OS. We then performed a multivariate analysis with the Cox regression model. Due to the missing data of ALK mutation status and M stage over 20%, they were not included in the multivariate analysis. The result indicated that CANT1 expression (*P* = 0.015, HR = 1.490 (95%CI [1.081-2.055])), T stage (*P* = 0.032, HR = 1.467(95% CI [1.034-2.081])) and pathological stage (*P* = 0.001, HR = 2.290 (95% CI [1.395-3.758])) were independently associated with worse OS.

**Table 4:** Univariate (a) and multivariate (b) Cox regression model of prognosis in patients with lung adenocarcinoma

Clinicopathologic variable	Total (N)	HR (95% CI)	*P*-value
a.			
Gender (Male vs. Female)	502	0.970(0.724-1.298)	0.837
Age (> 70 vs. <=70)	502	1.464(1.083-1.979)	0.013
Number pack years smoked (> 40 vs. <=40)	502	0.870(0.627-1.207)	0.405
EGFR mutation status (Yes vs. No)	272	1.261(0.800-1.987)	0.319
KRAS mutation status (Yes vs. No)	308	1.253(0.782-2.007)	0.348
ALK mutation status (Yes vs. No)	242	1.655(0.925-2.961)	0.090
T stage (T2&T3&T4 vs. T1)	502	1.666(1.182-2.347)	0.004
N stage (N2&N3 vs. N0&N1)	502	2.265(1.584-3.239)	< 0.001
M stage (M1 vs. M0)	502	2.143(1.251-3.672)	0.006
Pathologic stage (Stage III/IV vs. Stage I/II)	502	2.609(1.911-3.561)	< 0.001
CANT1 (High vs. Low)	502	1.547(1.143-2.094)	0.005
b.			
Age (> 70 vs. <=70)		1.341(0.984-1.827)	0.063
T stage (T2&T3&T4 vs. T1)		1.467(1.034-2.081)	0.032
N stage (N2&N3 vs. N0&N1)		0.881(0.500-1.550)	0.660
Pathologic stage (Stage III/ IV vs. Stage I/II)		2.290(1.395-3.758)	0.001
CANT1 (High vs. Low)		1.490(1.081-2.055)	0.015

*HR* Hazard ratio, *CI* Confidence interval, *EGFR* Epithelial growth factor receptor, *KRAS* Kirsten rat sarcoma viral oncogene, *ALK* Anaplastic lymphoma kinase, *T* Tumor, *N* Regional lymph node, *M* Metastasis, *CANT1* Calcium-activated nucleotidase 1

### Development of a prognostic model based on the expression of CANT1 and clinical factors

**Fig. 3:**
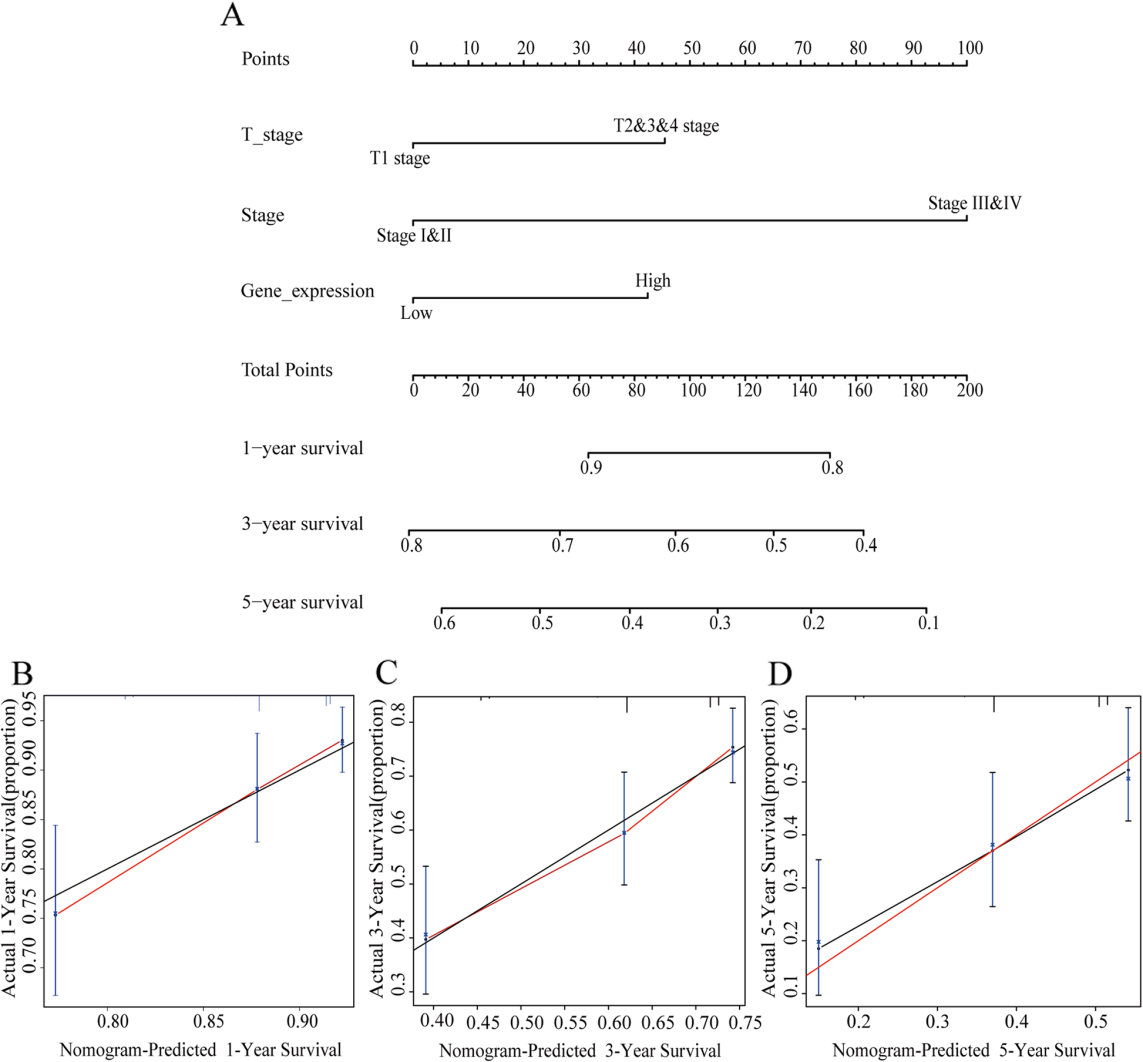
Nomogram and calibration curve for predicting the probability of OS for LA patients. **A** A nomogram that integrates CANT1 and other prognostic factors in LA from TCGA data; **B**-**D** The calibration curve of the nomogram in LA from TCGA data. OS, overall survival; LA, lung adenocarcinoma; CANT1, calcium-activated nucleotidase 1; TCGA, The Cancer Genome Atlas

A nomogram integrating the expression of CANT1, T stage, and pathological stage was constructed in our study (Fig. 3A). A worse prognosis was represented by a higher total number of points on the nomogram. The C-index was 0.64 based on 1000 bootstrap replicates for the nomogram. The deviation correction line in the calibration plot was close to the ideal curve, showing that the prediction result is in good agreement with the observation results (Fig. 3B-D).

### CANT1-related function enrichment obtained by Metascape

**Fig. 4:**
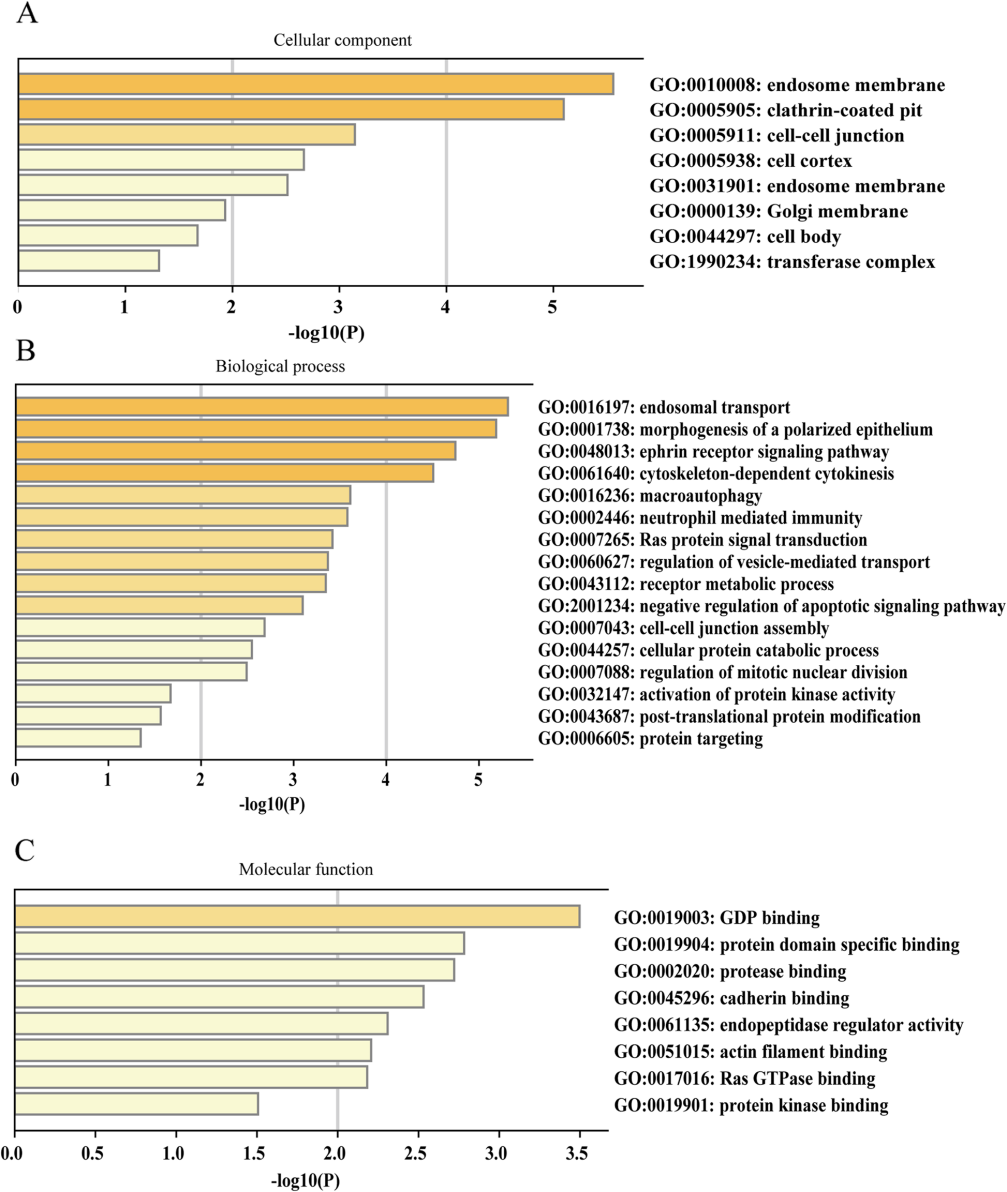
Functional enrichment of CANT1 in LA. **A**-**C** GO enrichment analysis of CANT1 and its co-expression genes in Metascape. The GO enriched terms are colored by *P*-value, where terms containing more genes tend to have a more significant *P*-value. LA, lung adenocarcinoma; GO, Gene ontology; CANT1, calcium-activated nucleotidase 1

**Fig. 5:**
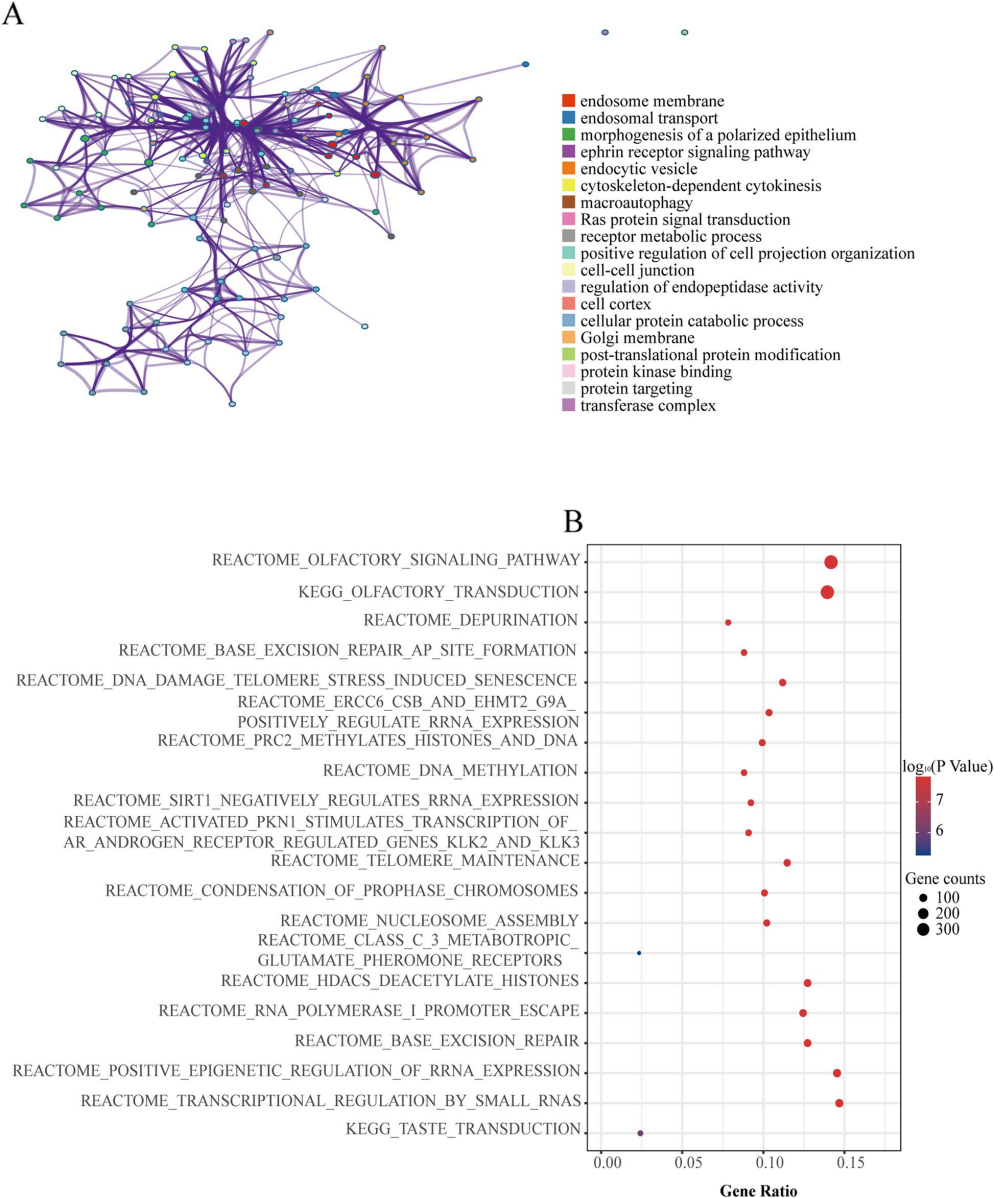
GO and GSEA functional enrichment of CANT1 in LA. **A** Network of GO enriched terms colored by *P*-values, where terms containing more genes tend to have more significant *P*-values. An interactive network of the top 19 enrichment terms. It is colored by cluster-ID. Distinct colors are various enrichment pathways of CANT1 correlated genes; **B** The top 20 pathways were differentially enriched in CANT1 related LA. The enrichment plot was obtained from the GSEA analysis. GO, Gene ontology; GSEA, gene set enrichment analysis; CANT1, calcium-activated nucleotidase 1; LA, lung adenocarcinoma

The GO enrichment items were engaged in several functional groups (Fig. 4A-C): cellular component (8 items), biological process (16 items), and molecular function (8 items). The cellular components for CANT1 and its most similar genes were mainly involved in cell-cell junction, cell cortex, and cell body. The biological processes were enriched in cytoskeleton-dependent cytokinesis, neutrophil-mediated immunity, Ras protein signal transduction, apoptotic signaling pathway, and mitotic nuclear division. The molecular functions were mainly engaged in GDP binding and Ras GTPase binding. Figure 5A showed that an interactive network of the top 19 enrichment terms. It is colored by cluster-ID. Distinct colors are various enrichment pathways of CANT1 correlated genes.

### CANT1-related signaling pathways obtained by GSEA

The TCGA RNA-seq data was used to compare CANT1-high and CANT1-low groups’ gene expression in LA. Finally, there are 19,479 differentially expressed genes were identified between the CANT1-high group and CANT1-low group. In order to gain further insight into the biologic pathways involved in LA by the expression level of CANT1, all the differentially expressed genes were analyzed by GSEA. The significantly enriched signaling pathways, mainly including depurination, DNA methylation, and DNA damage telomere stress-induced senescence are enriched based on normalized enrichment score (NES), adjusted *P*-value, and FDR value (Fig. 5B).

### Correlation between CANT1 expression and methylation

**Fig. 6:**
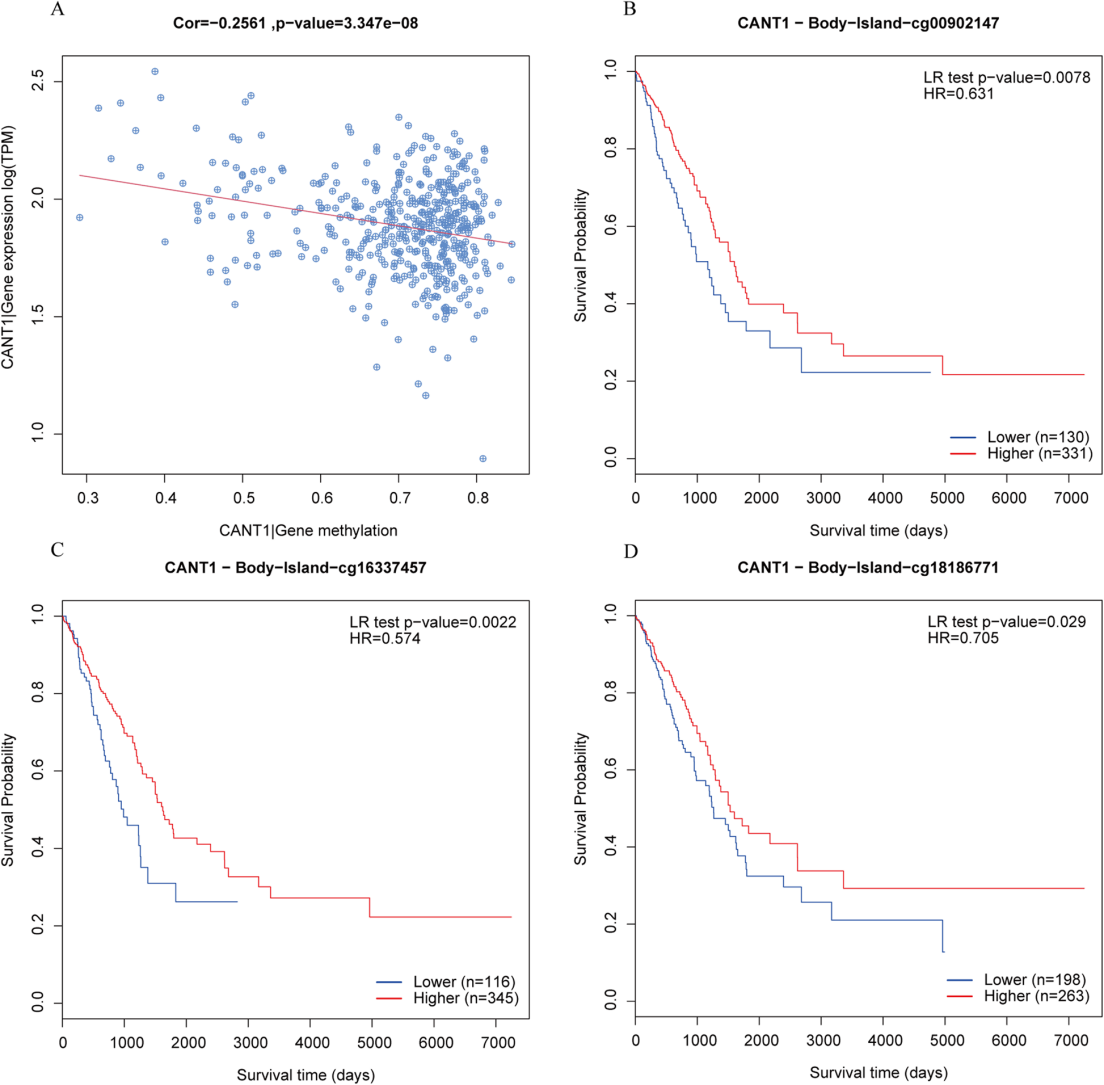
The methylation of CANT1 in LA. **A** The correlation between the methylation level and the CANT1 expression; **B**-**D** The Kaplan-Meier survival of the promoter methylation of CANT1. CANT1, calcium-activated nucleotidase 1; LA, lung adenocarcinoma

Figure 6A showed that the methylation level was significantly negatively correlated with CANT1 expression with Spearman up to − 0.2561 (*P* < 0.001). The effect of hypomethylation level and the expression of CANT1 on prognosis in LA was obtained from MethSurv. We discovered that 3 CpG sites located on the CpG island indicated a poor prognosis, including cg00902147, cg16337457, and cg18186771 (Fig. 6B-D).

### The expression levels of CANT1 in LAC lines

**Fig. 7:**
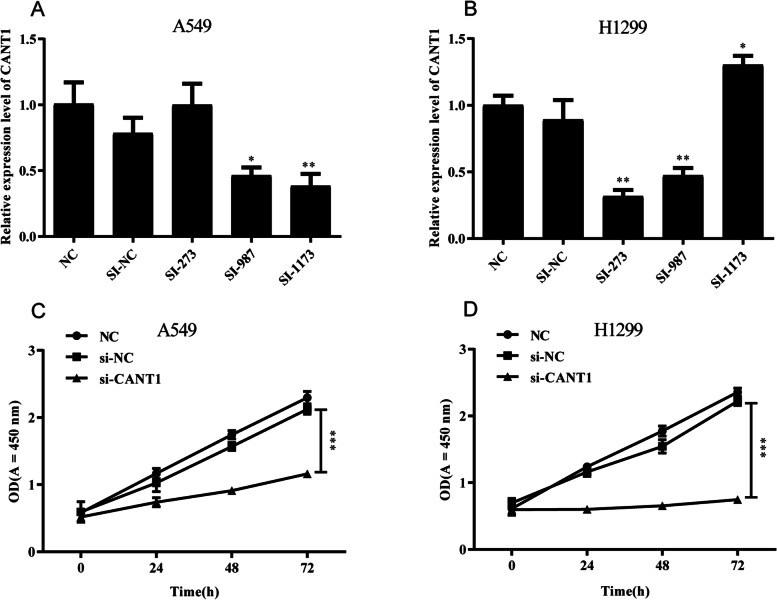
siRNAs effect examination and cell proliferation capacity comparison. **A**, **B** qRT-PCR showed that the inhibitory efficacy in A549 and H1299 cells transfected with NC, si-NC, si-273, si-987, and si-1173; **C**, **D** CCK8 assay showed that the proliferation capacity of A549 and H1299 cells in the NC group, si-NC group, and si-CANT1 group. (*P < 0.05, **P < 0.01, ***P < 0.001). siRNA, small interfering RNAs; qRT-PCR, quantitative real-time polymerase chain reaction; NC, negative control; CCK8, cell counting kit-8; CANT1, calcium-activated nucleotidase 1

We constructed A549 and H1299 cells by endogenously knocking down CANT1 by transfection of specific siRNAs, named as A549 si-CANT1 and H1299 si-CANT1. The expression of CANT1 was silenced by three siRNAs, as result, the qRT-PCR assay showed that si-1173 and si-273 had the best silencing effect among the three siRNAs against CANT1 in A549 and H1299 cell lines, respectively (Fig. 7A-B). The results indicated that si-1173 and si-273 were the most effective to inhibit the expression of CANT1 in A549 and H1299 cell lines, respectively. Therefore, they were selected in subsequent experiments.

### Cell counting kit-8 (CCK-8) and cell invasion experiments

**Fig. 8:**
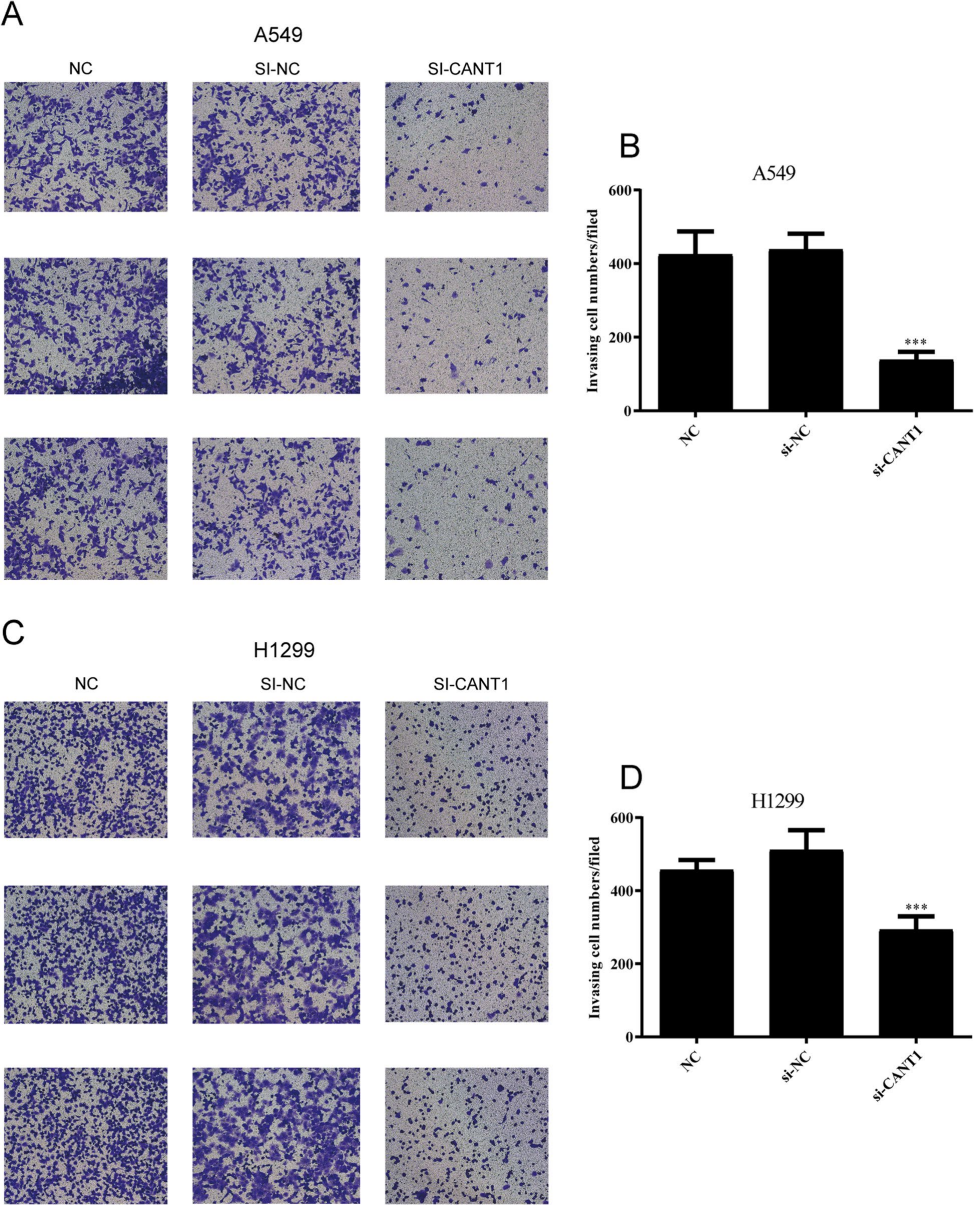
Knockdown of CANT1 inhibited cell invasion in LACs. **A** Invasion assay was performed to measure the invasive capacity of A549 cells after CANT1 knockdown; **B** The quantitative result was illustrated for A549 cells; **C** Invasion assay was performed to measure the invasive capacity of H1299 cells after CANT1 knockdown; **D** The quantitative result was illustrated for H1299 cells. (***P < 0.001 compared with NC group and si-NC group). CANT1, calcium-activated nucleotidase 1; LACs, lung adenocarcinoma cells; NC, negative control

To explore the effects of CANT1 on LACs’ proliferation and invasion, a series of in vitro experiments were carried out. The results of CCK-8 assays showed significant decline in the proliferation of A549 and H1299 cells after the knockdown of CANT1 (*P* < 0.001) (Fig. 7C-D). Transwell assay was conducted to evaluate the effects of CANT1 on cell invasion. CANT1 knockdown in A549 and H1299 cells remarkably inhibited the invasion ability (*P* < 0.001) (Fig. 8).

### Flow cytometry experiment

**Fig. 9:**
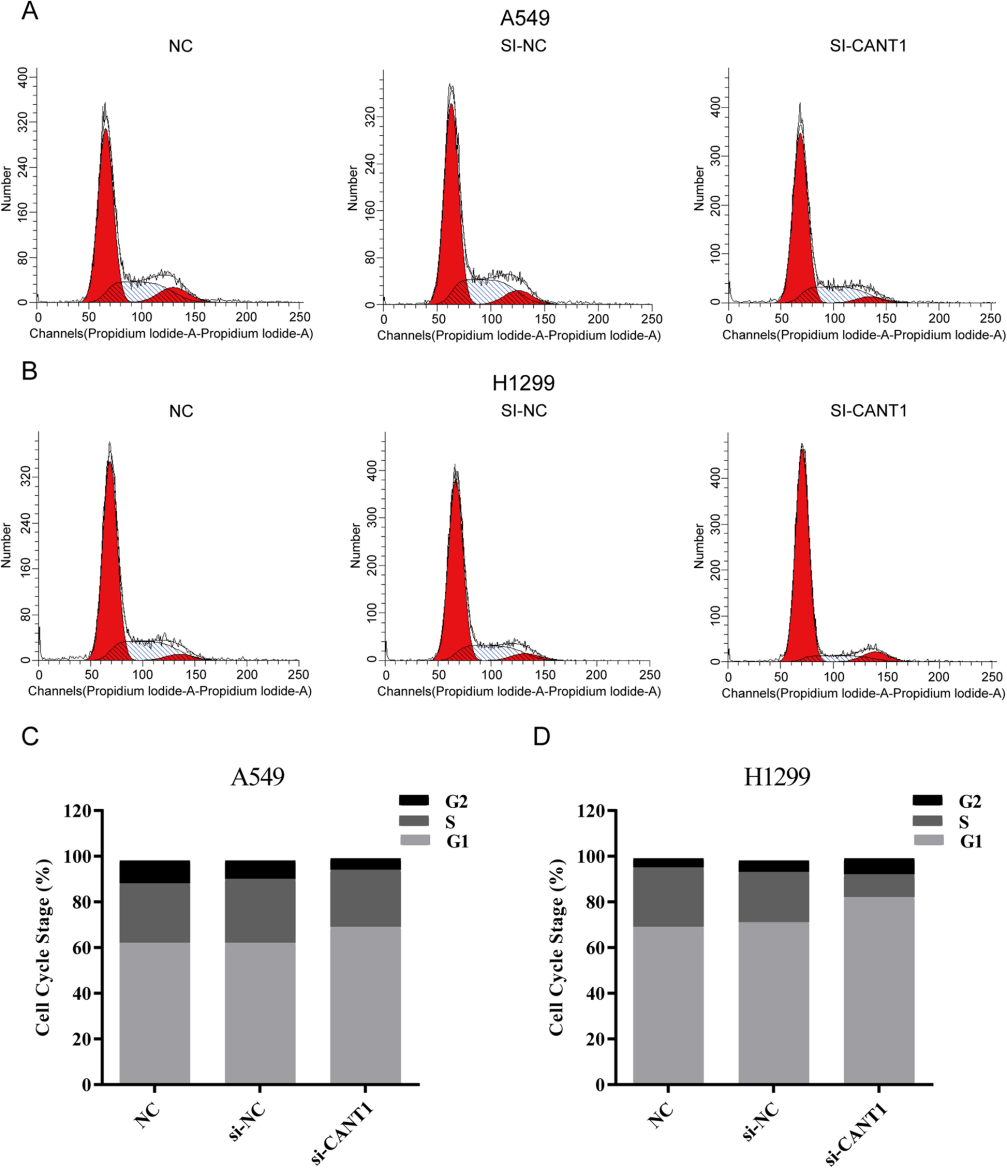
Cell cycle distribution in CANT1 knockdown cells. **A**, **B** Flow cytometry analysis of Propidium Iodide (PI) stained populations for cell cycle between NC group, si-NC group, and si-CANT1group in A549 and H1299 cells; **C**, **D** Distribution of G1, S, and G2 phase between NC group, si-NC group, and si-CANT1 group in A549 and H1299 cells. CANT1, calcium-activated nucleotidase 1; PI, Propidium Iodide; NC, negative control

To better understand the mechanisms of cell proliferation inhibited, the percentages of cells in different phases of the cell cycle were analyzed by flow cytometry. A significant increase in the G1 phase was observed in the si-CANT1 group (69.35 and 82.16%), compared with the NC group and si-NC group (62.41 and 69.65%, 62.43% and 71.66, respectively) in A549 and H1299 cells. Cell cycle was arrest in the G1 phase in the Si-CANT1 group, resulting in less cells entering the S phase (Fig. 9). Summarized, these results confirmed that knockdown of CANT1 could significantly inhibit the malignant phenotypes of LACs.

## Discussion

Lung adenocarcinoma (LA) accounts for almost half of lung cancers and patients with advanced LA have a very low survival rate [26]. Despite advances and efforts in understanding LA biology in recent years, the functional mechanisms of LA carcinogenesis remain elusive. This research aimed to identify gene with potential prognostic relevance. Up to now, there is no evidence that CANT1 relates to the occurrence and development of LA. Nevertheless, the role of CANT1 has been confirmed in other cancers, including clear cell renal cell carcinoma (ccRCC) [14] and prostate cancer [13]. To the best of our knowledge, it is the rare study to demonstrate the functional impact of CANT1 in LA.

Several studies have reported the prognostic significance of CANT1 for some cancers. High CANT1 expression was related to having a poor overall survival (OS) rate (*P* < 0.05) in ccRCC [14].The prognostic analysis was also carried out in our study. High expression of CANT1 was correlated with poor prognosis of LA in T2 to T4, N1 to N3, and stage II to IV. As shown above, our study suggested that CANT1 is a prognostic biomarker in LA. High expression of CANT1 indicated a worse survival rate.

In this study, GO enrichment analysis including Ras protein signal transduction, Ras GTPase binding, mitotic nuclear division, apoptotic signaling pathway, and cytoskeleton-dependent cytokinesis. These results suggested that CANT1 is related to cell division and growth, further supporting its relationship with tumorigenesis. Other enrichment results are related to intercellular recognition, interaction, and metabolism, which need to be confirmed by further study. GSEA analysis including DNA methylation, DNA damage telomere stress-induced senescence, and depurination. Evan et al. indicated that excessive accumulation of cells in cancer cells results from excessive cell proliferation and inadequate apoptosis. The inactivation of pro-apoptotic genes and the increased expression or activity of anti-apoptotic proteins lead to apoptosis deficiency [27]. Depurination is a process in which purines are lost in nucleic acids due to energy absorption. It participated in maintaining cell metabolism, which may affect cell growth. DNA depurination was related to the occurrence and development of cancer [28]. The previous study had also shown that CANT1 was involved in the therapy of purine and pyrimidine antimetabolites in cancer [29].

Several studies have demonstrated that the initiation and progression of tumors can be facilitated by epigenetic and genomic alterations [30], such as DNA methylation [31]. Despite many mechanisms contributing to elevated gene expression, DNA methylation is one of the most common mechanisms. Sato et al. indicated that high gene expression induced by hypomethylation was associated with poor prognosis in NSCLC [32, 33]. Through our analysis, we found that hypomethylation is correlated with the high expression level of CANT1. Our studies also showed that the hypomethylation level was associated with poor prognosis in LA patients. Therefore, CANT1 expression may be epigenetically regulated by DNA hypomethylation and has prognostic significance for LA. This result suggested that the hypomethylation of CANT1 may be associated with the high expression of CANT1.

To further confirm the biological mechanism of CANT1, we carried out a series of in vitro experiments in A549 and H1299 cells. Our experimental results indicated that knockdown of CANT1 resulted in decreased cell proliferation, invasion, and G1 phase cell-cycle arrest in LACs, which is consistent with our bioinformatics prediction. Based on the above results, CANT1 may take an active part in cell growth and division in LA and leading to the occurrence and development of LA.

Although the present study enhanced our understanding of the relationship between CANT1 and LA, our study had some limitations. First, our research was validated by in vitro experiments, we did not perform relevant experiments in vivo. Additionally, our investigations into the role of CANT1 in the tumor were based on the TCGA and GEO databases, which lacks verification from our clinical samples. Finally, we also cannot clearly investigate the direct mechanisms of CANT1 involved in the occurrence and development of LA. Our research has indicated the role of CANT1 in the metastasis of LA cells. Herein the results have shown that the CANT1 plays an important role in the invasion and migration of LA cells. It showed that the overexpression of CANT1 caused significant increase in the invasion and migration of LA cells, indicative of the role of CANT1 in LA cell metastasis. Although both CANT1 siRNA have interference effects, we have verified the most effective one. Nonetheless, it is fairly important to use one more CANT1 siRNA for the important experiments. At present, our research is mainly based on bioinformatics analysis and preliminary cell experiments to explore the role of CANT1 in LA, so in our future plan, we will further explore CANT1-mediated downstream pathways or molecules, so as to clarify the specific mechanism of CANT1 affecting the malignant phenotype of LA. It might be interesting to study whether the CANT1 gene may affect the biological function and mechanism of LA. Anyhow, a large number of samples and comprehensive studies are still required to strengthen the hypothesis that CANT1 is a prognostic biomarker.

## Conclusion

In conclusion, the present study suggested that CANT1 may serve as a potential prognostic biomarker in patients with LA. High CANT1 expression and promoter demethylation was associated with worse outcome. Finally, in vitro experiments verified the biological functions and behaviors of CANT1 in LACs. The specific mechanisms about knockdown of CANT1 expression inhibited the growth of LACs still need further research.

## Data Availability

The data that support the findings of this study are available from the corresponding author upon reasonable request.

## Abbreviations

CANT1: Calcium-activated nucleotidase 1
TCGA: The Cancer Genome Atlas
GEO: Gene Expression Omnibus
LA: Lung adenocarcinoma
NSCLC: Non-small cell lung cancer
LACs: Lung adenocarcinoma cells
GO: Gene ontology
GSEA: Gene set enrichment analysis
OS: Overall survival
UDP: Uridine triphosphate
ccRCC: Human clear cell renal cell carcinoma
DEGs: Differentially expressed genes
HPA: Human protein atlas
C-index: Concordance index
MSigDB: Molecular signatures database
ssGSEA: Single-sample gene set enrichment analysis
HR: Hazard ratio
CI: Confidence interval
FDR: False discovery rate
NES: Normalized enrichment score
FBS: Fetal bovine serum
siRNAs: Small interfering RNAs
NC: Negative control
qRT-PCR: Quantitative real-time polymerase chain reaction
CCK-8: Cell counting kit-8
PBS: Phosphate buffer saline
RNase: Ribonuclease
PI: Propidium Iodide
EGFR: Epithelial growth factor receptor
KRAS: Kirsten rat sarcoma viral oncogene
ALK: Anaplastic lymphoma kinase
T: Tumor
N: Regional lymph node
M: Metastasis

## References

[1] Siegel RL, Miller KD, Jemal A (2020). Cancer statistics, 2020. CA Cancer J Clin.

[2] Bray F, Ferlay J, Soerjomataram I, Siegel RL, Torre LA, Jemal A (2018). Global cancer statistics 2018: GLOBOCAN estimates of incidence and mortality worldwide for 36 cancers in 185 countries. CA Cancer J Clin.

[3] She J, Yang P, Hong Q, Bai C (2013). Lung cancer in China: challenges and interventions. Chest.

[4] Zheng M (2016). Classification and pathology of lung cancer. Surg Oncol Clin N Am.

[5] Zhao Y, Varn FS, Cai G, Xiao F, Amos CI, Cheng C (2018). A P53-deficiency gene signature predicts recurrence risk of patients with early-stage lung adenocarcinoma. Cancer Epidemiol Biomark Prev.

[6] Travis WD, Brambilla E, Nicholson AG, Yatabe Y, Austin JHM, Beasley MB, Chirieac LR, Dacic S, Duhig E, Flieder DB, Geisinger K, Hirsch FR, Ishikawa Y, Kerr KM, Noguchi M, Pelosi G, Powell CA, Tsao MS, Wistuba I, W. H. O. Panel (2015). The 2015 World Health Organization classification of lung tumors: impact of genetic, clinical and radiologic advances since the 2004 classification. J Thorac Oncol.

[7] Heist RS, Engelman JA (2012). SnapShot: non-small cell lung cancer. Cancer Cell.

[8] Uramoto H, Tanaka F (2014). Recurrence after surgery in patients with NSCLC. Transl Lung Cancer Res.

[9] Martinez-Terroba E, Behrens C, de Miguel FJ, Agorreta J, Monso E, Millares L, Sainz C, Mesa-Guzman M, Perez-Gracia JL, Lozano MD, Zulueta JJ, Pio R, Wistuba II, Montuenga LM, Pajares MJ (2018). A novel protein-based prognostic signature improves risk stratification to guide clinical management in early-stage lung adenocarcinoma patients. J Pathol.

[10] Smith TM, Hicks-Berger CA, Kim S, Kirley TL (2002). Cloning, expression, and characterization of a soluble calcium-activated nucleotidase, a human enzyme belonging to a new family of extracellular nucleotidases. Arch Biochem Biophys.

[11] Climente-Gonzalez H, Porta-Pardo E, Godzik A, Eyras E (2017). The functional impact of alternative splicing in cancer. Cell Rep.

[12] Revil T, Shkreta L, Chabot B (2006). Pre-mRNA alternative splicing in cancer: functional impact, molecular mechanisms and therapeutic perspectives. Bull Cancer.

[13] Gerhardt J, Steinbrech C, Buchi O, Behnke S, Bohnert A, Fritzsche F, Liewen H, Stenner F, Wild P, Hermanns T, Muntener M, Dietel M, Jung K, Stephan C, Kristiansen G (2011). The androgen-regulated Calcium-Activated Nucleotidase 1 (CANT1) is commonly overexpressed in prostate cancer and is tumor-biologically relevant in vitro. Am J Pathol.

[14] Liu X, Yang Z, Luo X, Luo J, Fu W, Fang Z, Xia D, Li L, Xu J (2019). Calcium-activated nucleotidase 1 silencing inhibits proliferation, migration, and invasion in human clear cell renal cell carcinoma. J Cell Physiol.

[15] Singh A, Kim OH, Iida A, Park WY, Ikegawa S, Kapoor S (2015). A novel CANT1 mutation in three Indian patients with Desbuquois dysplasia Kim type. Eur J Med Genet.

[16] Balasubramanian K, Li B, Krakow D, Nevarez L, Ho PJ, Ainsworth JA, Nickerson DA, Bamshad MJ, Immken L, Lachman RS, Cohn DH (2017). MED resulting from recessively inherited mutations in the gene encoding calcium-activated nucleotidase CANT1. Am J Med Genet A.

[17] Shan L, Zhou X, Liu X, Wang Y, Su D, Hou Y, Yu N, Yang C, Liu B, Gao J, Duan Y, Yang J, Li W, Liang J, Sun L, Chen K, Xuan C, Shi L, Wang Y, Shang Y (2016). FOXK2 elicits massive transcription repression and suppresses the hypoxic response and breast cancer carcinogenesis. Cancer Cell.

[18] Matsuda A, Suzuki Y, Honda G, Muramatsu S, Matsuzaki O, Nagano Y, Doi T, Shimotohno K, Harada T, Nishida E, Hayashi H, Sugano S (2003). Large-scale identification and characterization of human genes that activate NF-kappaB and MAPK signaling pathways. Oncogene.

[19] Asplund A, Edqvist PH, Schwenk JM, Ponten F (2012). Antibodies for profiling the human proteome-the human protein atlas as a resource for cancer research. Proteomics.

[20] Uhlen M, Fagerberg L, Hallstrom BM, Lindskog C, Oksvold P, Mardinoglu A, Sivertsson A, Kampf C, Sjostedt E, Asplund A, Olsson I, Edlund K, Lundberg E, Navani S, Szigyarto CA, Odeberg J, Djureinovic D, Takanen JO, Hober S, Alm T, Edqvist PH, Berling H, Tegel H, Mulder J, Rockberg J, Nilsson P, Schwenk JM, Hamsten M, von Feilitzen K, Forsberg M, Persson L, Johansson F, Zwahlen M, von Heijne G, Nielsen J, Ponten F (2015). Proteomics. Tissue-based map of the human proteome. Science.

[21] Tang Z, Li C, Kang B, Gao G, Li C, Zhang Z (2017). GEPIA: a web server for cancer and normal gene expression profiling and interactive analyses. Nucleic Acids Res.

[22] Zhou Y, Zhou B, Pache L, Chang M, Khodabakhshi AH, Tanaseichuk O, Benner C, Chanda SK (2019). Metascape provides a biologist-oriented resource for the analysis of systems-level datasets. Nat Commun.

[23] Mootha VK, Lindgren CM, Eriksson KF, Subramanian A, Sihag S, Lehar J, Puigserver P, Carlsson E, Ridderstrale M, Laurila E, Houstis N, Daly MJ, Patterson N, Mesirov JP, Golub TR, Tamayo P, Spiegelman B, Lander ES, Hirschhorn JN, Altshuler D, Groop LC (2003). PGC-1alpha-responsive genes involved in oxidative phosphorylation are coordinately downregulated in human diabetes. Nat Genet.

[24] Subramanian A, Tamayo P, Mootha VK, Mukherjee S, Ebert BL, Gillette MA, Paulovich A, Pomeroy SL, Golub TR, Lander ES, Mesirov JP (2005). Gene set enrichment analysis: a knowledge-based approach for interpreting genome-wide expression profiles. Proc Natl Acad Sci U S A.

[25] Modhukur V, Iljasenko T, Metsalu T, Lokk K, Laisk-Podar T, Vilo J (2018). MethSurv: a web tool to perform multivariable survival analysis using DNA methylation data. Epigenomics.

[26] Travis WD, Brambilla E, Noguchi M, Nicholson AG, Geisinger KR, Yatabe Y, Beer DG, Powell CA, Riely GJ, Van Schil PE, Garg K, Austin JH, Asamura H, Rusch VW, Hirsch FR, Scagliotti G, Mitsudomi T, Huber RM, Ishikawa Y, Jett J, Sanchez-Cespedes M, Sculier JP, Takahashi T, Tsuboi M, Vansteenkiste J, Wistuba I, Yang PC, Aberle D, Brambilla C, Flieder D, Franklin W, Gazdar A, Gould M, Hasleton P, Henderson D, Johnson B, Johnson D, Kerr K, Kuriyama K, Lee JS, Miller VA, Petersen I, Roggli V, Rosell R, Saijo N, Thunnissen E, Tsao M, Yankelewitz D (2011). International association for the study of lung cancer/american thoracic society/european respiratory society international multidisciplinary classification of lung adenocarcinoma. J Thorac Oncol.

[27] Evan GI, Vousden KH (2001). Proliferation, cell cycle and apoptosis in cancer. Nature.

[28] Cavalieri E, Saeed M, Zahid M, Cassada D, Snow D, Miljkovic M, Rogan E (2012). Mechanism of DNA depurination by carcinogens in relation to cancer initiation. IUBMB Life.

[29] Fridley BL, Batzler A, Li L, Li F, Matimba A, Jenkins GD, Ji Y, Wang L, Weinshilboum RM (2011). Gene set analysis of purine and pyrimidine antimetabolites cancer therapies. Pharmacogenet Genomics.

[30] Kanda M, Sugimoto H, Kodera Y (2015). Genetic and epigenetic aspects of initiation and progression of hepatocellular carcinoma. World J Gastroenterol.

[31] Pan Y, Liu G, Zhou F, Su B, Li Y (2018). DNA methylation profiles in cancer diagnosis and therapeutics. Clin Exp Med.

[32] Sato T, Soejima K, Arai E, Hamamoto J, Yasuda H, Arai D, Ishioka K, Ohgino K, Naoki K, Kohno T, Tsuta K, Watanabe S, Kanai Y, Betsuyaku T (2015). Prognostic implication of PTPRH hypomethylation in non-small cell lung cancer. Oncol Rep.

[33] Noguera-Ucles JF, Boyero L, Salinas A, Cordero Varela JA, Benedetti JC, Bernabe-Caro R, Sanchez-Gastaldo A, Alonso M, Paz-Ares L, Molina-Pinelo S (2020). The roles of imprinted SLC22A18 and SLC22A18AS gene overexpression caused by promoter CpG island hypomethylation as diagnostic and prognostic biomarkers for non-small cell lung cancer patients. Cancers (Basel).

